# Infant feeding choices among Panamanian mothers: A qualitative study

**DOI:** 10.1002/fsn3.3535

**Published:** 2023-07-06

**Authors:** Danae De La Cruz, Richard Philip Lee, Justine Gallagher

**Affiliations:** ^1^ Ministry of Health Panama City Republic of Panama; ^2^ Faculty of Health & Life Sciences Northumbria University, Coach Lane Campus (West) Newcastle upon Tyne UK

**Keywords:** breastfeeding, infant feeding decisions, Panama, qualitative research, social factors

## Abstract

Infant and child nutrition practices are among the most critical determinants of infant health and breastfeeding is considered the gold standard of infant feeding. Despite extensive public health interventions to promote breastfeeding, its prevalence has decreased in recent years in Panama, particularly in urban settings. There has been a nearly 20% drop in breastfeeding in the 10 years leading to 2020. Current literature often fails to elucidate the factors underpinning Panamanian mothers' decision making in relation to breastfeeding. This article explores the experiences, views, and decision making related to infant feeding choices of mothers in Panama City. The study used a qualitative approach, involving online semistructured interviews with seven participants. Utilizing the socioecological model enabled an understanding of the influence of the various, nested levels of a mother's social environment on behaviors and practices. Five themes were developed following analysis: “practical, bodily, and emotional challenges”; “workplace influences”; “family and friends’ support”; “the role of health care and healthcare professionals”; “the influence of social and cultural norms on decisions and practices.” The main barrier to breastfeeding was the lack of family support, especially from grandmothers. In contrast, private lactation consultation and partners' support were perceived as the best approaches for breastfeeding success, suggesting an urgent need for publicly available lactation support. This study demonstrates the importance of understanding the complexity of the social norms surrounding infant feeding, showing the challenges that mothers face in this process, and sheds light on the (public) interventions necessary to improve breastfeeding initiation and continuation.

## Graphical abstract

In this article we make a novel contribution to the literature, presenting (we believe for the first time) the experiences of Panamanian mothers in regard to their infant feeding decisions. This qualitative study opens a new avenue of research to develop our understanding of the factors that influence infant feeding decisions by Panamanian mothers and demonstrates the importance of understanding the complexity of the social norms surrounding infant feeding. The study concludes that current Panamanian breastfeeding policies do not address the impact of family and friends on the decisions of Panamanian mothers. Further is study is also required of the impact workplace policies, routines and support make to feeding decisions and international comparative analysis may prove a fruitful in this regard.

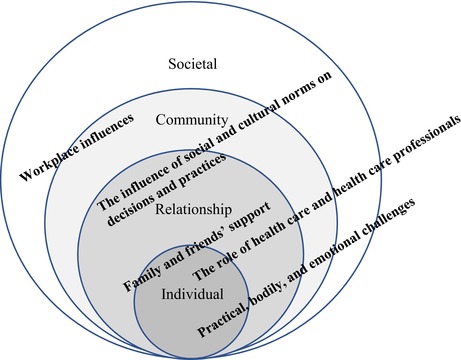

## 1 INTRODUCTION

### 1.1 Infant nutrition and breastfeeding practices

Infant and child nutrition practices are among the most critical determinants of infant health (Abekah‐Nkrumah et al., 2020) and breastfeeding is considered the gold standard of infant feeding (Benoit et al., 2016). Exclusive breastfeeding (EBF) in the first 6 months of life is an effective measure to fight against the double burden of malnutrition, to improve food security and reduce health inequalities (Rodríguez Diaz et al., 2017). The World Health Organization (WHO) indicates that optimal feeding practices should include exclusively breastfeeding (with no other food than breast milk, no other milk, or drinks including water) in the first 6 months of life; to continue breastfeeding up to 2 years of age with the introduction of complementary food (Rodríguez‐Vazquez et al., 2020). Although the prevalence of breastfeeding globally has increased in recent decades, it is not enough to meet the objectives set by international organizations (Walters et al., 2016).

Breastfeeding is no longer considered the norm for many women (Rollins et al., 2016) and as a result, only 40% of children worldwide are breastfed through the first 6 months of life (Rahman & Akter, 2019). These rates are highly variable between countries and within countries, as well as between women in the same society with different cultural and socioeconomic characteristics (Sheehan et al., 2010). The factors that influence fluctuations in breastfeeding rates include changes in women's role in societies, the adoption of different healthcare practices after delivery (e.g., the length of hospital‐based maternity care), and the aggressive marketing of the infant food industry (Cattaneo, 2012). In the last decade, breastfeeding rates have improved in most countries in Latin America, however, changes in breastfeeding rates have shown a decrease in subgroups whose infants are most at risk for early death, described by Bueno‐Gutierrez and Chantry (2015) as those mothers with lower levels of education and limited access to health care (and reflecting socioeconomic inequities and inequalities). In the Global Nutrition Report (2020), it is argued that Panama has made no progress in meeting the goals for maternal and young infant nutrition. Despite the benefits and extensive public health interventions to promote breastfeeding, its prevalence has decreased in recent years in Panama. According to the National Sexual and Reproductive Health Survey, exclusive breastfeeding rates of infants aged 0 to 5 months dropped from 42% in 2006 to 21.5% in 2016. These low rates of breastfeeding are more typically associated with well‐educated, working mothers who live in urban areas and give birth in hospitals (Rodríguez Diaz et al., 2017).

Instead of interpreting the decision to breastfeed as an individual issue that falls solely with the mother, the influence of social, environmental, and societal factors and processes should be considered as part of the analysis (Brown, 2017), in keeping with a socioecological approach (see Section 2.4). In this article, we identify the factors that affect feeding decisions and help fill this gap in the literature by giving a sense of what factors influence decisions of urban‐residing mothers in Panama City. There is a lack of qualitative research studies identifying factors that influence the infant feeding choices in this group. This study aims to fill a gap in the literature by exploring the experiences, views, and decision making of mothers from an urban metropolitan setting, in relation to infant feeding choices.

### 1.2 Key aims and messages

In this article we make a novel contribution to the literature, presenting (we believe for the first time) the experiences of mothers in Panama City with regard to their infant feeding decisions. This qualitative study opens a new avenue of research to develop our understanding of the factors that influence infant feeding decisions by Panamanian mothers and demonstrates the importance of understanding the complexity of breastfeeding practices by situating our analysis within the socioecological model. The study concludes that current Panamanian breastfeeding policies do not address the impact of family and friends in the decision of mothers on infant feeding choices. Further study of the impact workplace policies and routines and of the potential impact of publicly available lactation support is required, and international comparative analysis may prove fruitful in this regard.

## 2 METHODS

### 2.1 Setting and relevant context

Participants were recruited from Panama City, the capital of the Republic of Panama and the most urbanized city in Panama. Panama is a middle‐income country located in Central America with a population of about 4.5 million and whose official language is Spanish. If breastfeeding is analyzed demographically in Panama, mothers from rural areas (30%) are more likely to breastfeed than those from urban areas (16.6%) (Global Report, 2020). In terms of wealth, mothers from the lowest socioeconomic status presented the highest breastfeeding rates (38%), while middle class mothers presented the lowest (11%). Additionally, regarding educational level, women with none or primary education have higher rates of breastfeeding compared to mothers with secondary or higher education (Global Report, 2020).

### 2.2 Sampling strategy

The sample comprised seven Panamanian mothers residing in Panama City. The sample size was appropriate for an exploratory, qualitative study design (Creswell, 1998; Tracy, 2020) and generated data relevant to the main research questions, providing “information power” (Malterud et al., 2016). The study aimed to obtain detailed, in‐depth descriptions of mothers' experiences. All the study's participants spoke Spanish as their first language.

The participants were recruited via social media. The contact with participants was made after obtaining ethical approval from the Faculty of Health and Life Sciences Research Ethics Committee at the Northumbria University. Participants who were eligible for the study were mothers over 18 years old, primiparas or multiparas, whose child, at the time of the interview, was 2 years old or younger. Mothers of preterm infants and mothers of infants with existing feeding problems were excluded because of their potential negative effects on infant feeding decisions. The first seven mothers that met the inclusion criteria were invited to participate in the interview. After the initial contact, where the researcher introduced herself, a discussion was held about what participation in the study entailed. A convenient time and day were arranged with each mother to conduct the online interview.

### 2.3 Data collection

Between March and April 2021, semistructured interviews were conducted with each of the seven mothers about their experiences of the decision to whether breastfeed or formula feed. First, mothers were asked for demographic information on maternal occupation, marital status, and maternal and infant age. The purpose of the demographic questions was to provide a general description of the mothers' characteristics as a group and to seek a maximum variation sampling strategy with the inclusion of seven participants from different backgrounds within the sample of Panamanian mothers.

Interviews lasted between 30 and 45 min. Deploying semistructured interviews allowed the mothers to talk openly about factors that affected their decision making. Before starting the interview, verbal informed consent was obtained. As part of the informed consent process, permission to use a digital audio recorder was sought, along with an explanation of the implications of taking part in the study. Participants were informed of the study's purpose and advised that the interviewer did not intend to promote a specific feeding method. They were also aware of their rights as participants, including their right to withdraw from the study at any time. The mothers were asked to talk as freely as possible about their infant feeding experiences and respond to the open‐ended questions. Sample questions were: Can you tell me about your experience of feeding your child? When did you make the decision as to whether you would breastfeed or feed formula to your baby? The interviews were conducted in Spanish through ZOOM, an online video communication platform.

### 2.4 Data analysis

The voice recordings were transcribed verbatim. Each transcript was read multiple times, for accuracy. The interviews were analyzed through thematic analysis. Significant phrases that were related to the phenomenon under study (infant feeding practices and decision making) were coded and extracted and meanings formulated. Themes were compared with the transcripts, noting similarities and differences. The data were analyzed in the original language and then translated into English to ensure reliability and transparency. The researcher who carried out the primary analysis is Panamanian and a Spanish native speaker, allowing an understanding of the sociocultural context for a culturally sensitive translation and proper interpretation of the participants' interviews. The emergent analysis was discussed in several meetings with the second author to enhance validity, reliability, and coherency.

The use of a socioecological model enabled understanding of the influence of the social environment on behaviors. The model, first elaborated by Bronfenbrenner, includes five nested systems or levels; though there have been a variety of iterations and applications developed over time, often invoking fewer systems (Bueno‐Gutierrez & Chantry, 2015; Khasawneh, 2016; Kilanowski, 2017). The microsystem, or individual level, comprises the characteristics of the individual that shapes behaviors (in this case, breastfeeding intention, knowledge, and confidence). The mesosystem, or relationship level, comprises (again, in the case of this study) support from a partner, family, and friends or healthcare providers (which influence the individual's behavior). The exosystem, or community level, includes the physical environment and institutions (e.g., public places and spaces where breastfeeding might be performed). The community level can exert an indirect influence upon practices. The final level is macrosystem, or societal level, which comprises societal, cultural values and policy (to note, some iterations of the socioecological model, including Bronfenbrenner's, place changes over the life course within an additional level of the chronosystem).

## 3 RESULTS

### 3.1 Introduction

Seven mothers participated in the study. All participants were married or living with a full‐time partner. Six of the mothers were professional, and one was a stay‐home mother. Table 1 shows the feeding method at 6 months, maternal age, the infant's age at the time of the interview, working status of the mother, and place of birth of the child.

**TABLE 1:** Characteristics of the participants.

Participant	Feeding method	Maternal age	Age of the child	Working status	Place of birth of the child
1	EBF until 1.5 months, then breastfeeding with occasional formula. Exclusive formula from 4 months.	27	18 months	Self‐employed	Private hospital
2	EBF until 1.5 months. Formula feeding for 1 month and then exclusive breastfeeding.	26	15 months	Employed (part‐time)	Public hospital
3	EBF for 3 weeks, then introduced formula. Exclusive formula from 3 months.	30	10 months	Employed	Private hospital
4	EBF	33	24 months	Employed (working from home)	Private hospital
5	EBF	32	4 months	Employed (working from home)	Private hospital
6	EBF	28	3 months	Employed	Public hospital
7	BF with the introduction of water before 6 months.	24	15 months	Unemployed	Public hospital

Abbreviations: BF, breastfeeding; EBF, exclusive breastfeeding.

**FIGURE 1:**
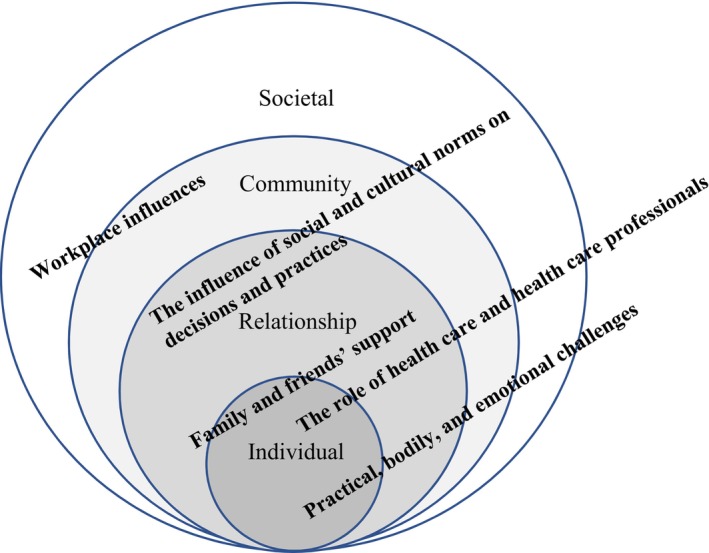
Themes mapped onto the socioecological model.

Our analysis produced five themes: practical, bodily, and emotional challenges; workplace influences; family and friends' support; the role of health care and healthcare professionals; the influence of social and cultural norms on decisions and practices. All five themes demonstrated the relevancy of the socioecological model to analyzing breastfeeding practices, in that connections between individual, social, community, and societal levels can be seen, to varying degrees, across the themes (these connections are discussed in more detail next). Figure 1 maps the main themes across the nested levels of the socioecological model:

All five themes have a relationship to the individual, but in Figure 1 we have sought to emphasize the significance of other nested levels. For example, the theme “workplace influences” is more strongly connected to the “community” and “societal” levels. We consider each theme in more detail in the following sections.

### 3.2 Practical, bodily and emotional challenges

All participants (P) had the intention to exclusively breastfeed for at least 6 months and achieved this for at least 3 weeks. When asked about their experiences, many mothers experienced practical, bodily, and emotional challenges with breastfeeding. They mentioned that in the first month, breastfeeding was very demanding, tiring, stressful, and painful:

P1. It is exhausting (*breastfeeding*), totally exhausting. I also had to go back to work, and the effort involved in breastfeeding is huge. Then it was easier to make the bottle at night.

P3. Breastfeeding feels uncomfortable; it wasn't so magical. It was difficult. Scab, bleeding, cracked nipple, that was basically why I decided that I couldn't do it anymore, because at one point I said, "I don't want him near me" (*referring to the baby*). When I introduced formula, it was definitely a relief; it is much more practical…. But I felt guilty.

On the other hand, for some women, with the help of a lactation consultant, these problems were solved within a few days:

P4. Even though I had a lactation coach, I had moments in which I believed that I would not be able to do it (*EBF*). It is stressful, and it is a lot of work that must be done, but I did it with a lot of love because I understand and believe that breast milk is the best food for him.

Loss of autonomy and associated feelings of isolation as a consequence of breastfeeding were common barriers described by mothers (reflecting the complex relationship between the individual and social levels of the socioecological model). Even those who were most committed to breastfeeding also mentioned that they felt the need to isolate when they had to breastfeed in public as “respect for others,” even when in front of the family:

P2. In restaurants, I had to go to a hidden corner, people passed by and looked at me. You feel bad because you have to isolate yourself, and you cannot share those moments with your family.

P3. When I had to breastfeed in front of my dad, I had to sit in the corner farthest from him. But it was mainly because I was uncomfortable, and he was uncomfortable. That felt a bit sad because I had to be far away, where nobody saw me, and not because someone told me to do that. You tacitly feel that you have to exclude yourself.

Such isolating practices were also an outcome of the community and physical environment in which mothers lived and worked (the third level of the socioecological model). The role of the community and physical environment is discussed in more detail in the theme “workplace influences.”

### 3.3 Workplace influences

Returning to work had a significant impact on infant feeding decisions and practices. Some mothers described how they had to use annual leave as an extension of maternity leave (in order to have more time with their baby) and most women returned to work when their babies were 3 months or less. Two mothers identified that returning to work was the main reason why they stopped breastfeeding, while one of the mothers (who worked in a Baby‐Friendly Hospital) mentioned that there was no space in her place of work with the right conditions for her to express milk. Our analysis also indicates that a lack of institutional support is a factor that interferes with the decisions of the mothers to breastfeed, with the consequential absence of lactation rooms, adequate maternity leave, or breastfeeding breaks. Five working mothers indicated that there was no hygienic/adequate space for them to express their milk. Such combination of inadequate physical space and regulatory conditions demonstrates the interconnectedness of two levels of the socioecological model in particular: the community and physical environment level and the societal level.

Mothers had different experiences related to support received from their coworkers. One participant mentioned that her coworkers were one of the most important parts of her support network:

P6. My co‐workers (*nutritionist*), made it easier for me to breastfeed at work. One is a lactation consultant, and the other has experience in breastfeeding too. For me, they have a lot of experience. They help a lot when I have doubts.

In contrast, one mother experienced negative reactions from coworkers about her decision.

P3. One of my co‐workers (*nutritionist*) stopped talking to me for about two days when she found out that I stopped breastfeeding. I couldn't talk about how I was feeding my baby without feeling that people in my work would judge me. I'm a nutritionist, and I work in a hospital.

For both situations, the individual and social levels of the socioecological model combine but can produce very different situations and experiences.

### 3.4 Family and friends' support

All the participants discussed receiving advice from their parents which went against the recommendations of the doctor and against their decision to breastfeed. The participants suggested that such advice is caused by a lack of knowledge about breastfeeding, affecting their parents' ability to give adequate support. Mothers and mothers‐in‐law were described as showing interest in infant feeding, but their involvement was not always considered to be supportive. Two mothers describe how they felt pressure to introduce artificial milk to their children in order to get more sleep:

P1. My mother‐in‐law kept telling me that I had to give her formula so that we could rest. She used to tell me that she gave her children formula to sleep through and we should do the same.

P2. My mom said to me, “you must give him formula because you need to sleep, look at you, you have to sleep, and if you give him formula, he will sleep all night.” I felt helpless.

Others stated that their own family made negative comments, making them feel insecure. The advice to introduce water and formula for the infant's health was also commonly mentioned:

P6. My mother told me to give him formula because I was not producing any breastmilk. I just had 28 hours of giving birth. I never thought of giving formula but imagine being told, “you don't have milk,” with all the changes in my body, that made me feel distrustful.

P7. My mother‐in‐law and my mother told me that I had to give him formula because breast milk does not fill him and that I had to give him water. But the paediatrician said the opposite. They insisted so much that I gave him water before six months. I did it because I wanted to avoid conflicts.

Even though women's experiences of support from their partners were varied, all seven mothers considered their partner to be one of the main supporters in their decision. Most of the participants were aware of the cost of artificial milk, and their partners recognized this as one of the benefits of breastfeeding. Many mothers also considered this when making infant feeding decisions. Some partners were not supportive of breastfeeding in public (asking them to cover up), while others were more supportive, seeing breastfeeding as “natural.” Those women whose partners asked them to cover up did not breastfeed more than 3 months.

All participants mentioned the need to feel supported and not be subjected to any prejudice regarding the feeding method they chose. Some mothers mentioned that they were going through a period of many changes, which made them vulnerable. Two other mothers stated that their own family and healthcare practitioners offered negative comments that made them feel insecure. A mother expressed doubt about her decision making when listening to a friend's advice:

P2. She said to me, “your life is going to change, you will be able to sleep more, I promise you, you have to give him formula. I was just like you, nothing will happen to him,” and I was very tempted, with a mastitis that was killing me, if it had not been for my husband, I would have abandoned breastfeeding that day.

As with interactions with coworkers under the previous theme, through these examples it is evident that the individual (micro) and social (meso) levels of the socioecological model are connected. Furthermore, aspects of the societal (macro) level, in particular cultural values, operate to influence the type of advice offered by some older family members.

### 3.5 The role of health care and healthcare professionals

Many women expressed dissatisfaction with professional advice and support regarding feeding options, reporting that the healthcare practitioner's advice was often impractical, inconsistent, and difficult to understand. Most of the participants indicated that their healthcare providers did not advise them on breastfeeding benefits and they had to seek information from different sources. It was suggested that healthcare practitioners gave contradictory information, for example, stating that mothers should breastfeed, but that they should resort to an easy and quick solution (formula feeding) when a problem arises.

P4. I seek information on how to breastfeed elsewhere because if it had been for my paediatrician, I would have given my baby formula. After the delivery, she made me the prescription for the formula, without even asking me.

All mothers who exclusively breastfed and continue to breastfeed for more than 6 months received the help of a private lactation consultant. None of the mothers reported having had antenatal education provided by the healthcare practitioner or healthcare centers. In one particular case, a mother (who also EBF) mentioned receiving antenatal education from a public healthcare center regarding feeding methods.

P4. Thanks to my lactation coach was that I could breastfeed. That help is essential. It is something that one should always contemplate when you are going to have a baby. If you do not have help, you will be lousy.

P3. I think what I missed doing was having a lactation consultant. I did not do it out of stubbornness. I felt that I needed guidance on how to do it so that it wouldn't hurt so much.

In terms of the socioecological model, this theme demonstrates how individual, social, and societal levels are nested, as participants discuss the interconnections between their own priorities, interactions with healthcare professionals and wider societal factors such as the availability of support and care and the resourcing (and training) of antenatal services.

### 3.6 The influence of social and cultural norms on decisions and practices

The first theme (“practical, bodily, and emotional challenges”) described some of actions taken by the participants when breastfeeding in public. In this final theme, we consider the impact of social and cultural norms specifically (and so seek to examine connections across the socioecological model, from individual to societal levels). Four of the mothers reported that breastfeeding in public was normal, and they see it as natural. They mentioned that they had not perceived that people were looking at them. Others express that they are not affected by being looked at by people. By contrast, those who switched to formula feeding perceived breastfeeding in public as uncomfortable.

P4. I feel that there are people who do not feel comfortable with you breastfeeding, but that does not matter to me. From the beginning, I decided not even to cover myself.

P3. The truth is that breastfeeding in public is uncomfortable. If you compare taking the breast out vs preparing formula, nobody stops to look at you twice. When you give a formula, nobody looks at you. It is much more accepted and normalised for someone to take out a bottle than to take out the breast.

On the other hand, one of the mothers mentioned that they had to cover themselves for breastfeeding in public and criticized those who did not cover.

P1. I didn't care about breastfeeding in public. Obviously, sometimes I had to put the blanket over the baby's face because we were eating in a public place, and I, because of modesty, so that people would not feel uncomfortable, I put the blanket on. But I had a colleague who was crazy. She pumps her milk in the middle of the office in front of everyone without covering herself. I'm not going to pump myself in front of the whole world, taking out my breasts if I don't have the baby.

With regard to the perception of pressure to breastfeed, the mothers had diverse views. Some commented that society puts a lot of pressure on mothers to breastfeed, and if you do not breastfeed you are judged to be a “bad mother.” Others mentioned the pressure they felt to introduce formula as a solution to the difficulties of motherhood. One mother noted a contradiction regarding breastfeeding in society. She suggested that there is a stigma for breastfeeding in public (i.e., not discussed and made visible), but, on social media, mothers are bombarded with information with “breast is the best,” and if you do not breastfeed, you are a bad mother. The interplay between social and cultural norms in the “corporeal world” and in social media interactions and information provision demonstrates the complex arrangement of individual, social, community (in particular different kinds of physical and virtual “space”), and societal levels.

## 4 DISCUSSION

This study provides unique insights into the infant feeding decisions and practices of mothers in Panama City. Regardless of how they decided to feed their children, it was found that they made this decision not in isolation but, as per the socioecological model, through the complex interplay of individual, social, community, and societal factors. These findings are supported by previous research that has shown that maternal decision making related to infant feeding is determined by a combination of factors and not solely by the mother's desires (Brown, 2017). Consistent with the socioecological model, the findings of this study suggests that a combination of individual, social, community (including the physical environment), and societal factors explain these mothers' infant feeding decisions and that the influence of these factors can vary according to the breastfeeding practice in question. In the setting where our participants were based – Panama City – it is important to note that breastfeeding rates can also be affected by migration from rural and indigenous areas to urban areas, through processes of acculturation and exposure to nontraditional practices (Rodríguez Diaz et al., 2017). By way of comparison, Hawkins et al. (2008), cited in Condon (2018), suggested that the rates of breastfeeding in migrant mothers who move to the United Kingdom decline once they have settled in a community. Condon (2018) suggested that this could not only be down to structural factors such as employment, but also due to “acculturation” as the mothers adopt the behaviors of the host society.

All the mothers in this study had the intention to breastfeed for more than 6 months, acknowledging the benefits of breastfeeding. However, their decision to sustain breastfeeding was affected by the practices evident in the five themes we discussed: practical, bodily, and emotional challenges; workplace influences; family and friends' support; role of health care and healthcare professionals; the influence of social and cultural norms on decisions and practices. This study shows that mothers who decided to introduce formula did so mainly because they did not know how to manage or solve the problems that arose with breastfeeding across these five themes. A study conducted by Fox et al. (2015) found that the lack of knowledge of how to solve sore nipples, mastitis, pain, and incorrect latch leads to early cessation of breastfeeding. Bartle and Harvey (2017) suggested that such issues could be addressed by educating the mother and with a good support system. These findings are consistent with those expounded in this study. Mothers who could establish breastfeeding also had problems related to breastfeeding management, which they managed to resolve. This difference in the management of problems and the outcome of the infant feeding decision could be due to the fact that these mothers had the help of a private lactation consultant (education) and the support of their partner at all times (good support system).

The time invested in breastfeeding and the embarrassment when breastfeeding in public contributed to the feeling of loss of independence and stigma. The mothers who introduced infant formula all commented that they felt uncomfortable breastfeeding in public and felt the need to cover or isolate themselves so that no one could see them, including family members. As reported in the literature, such feelings arise from the perception of the act of breastfeeding as socially unacceptable (Radzyminski & Callister, 2016). The demanding nature of EBF also contributed to the lack of time mothers had to carry out general activities, with almost no time for themselves. This lack of time was exacerbated when mothers had to return to work, with the demands of work and expressing milk concomitantly becoming unmanageable. Some mothers were placed in a situation where they had to return to work when their infant was only 6 weeks old. In addition, there was not always compliance by employers with requirements and guidance, such as mandatory breaks or appropriate place for expressing milk. These findings confirmed the critical role of environmental and policy level factors like work environments and workplace policies in supporting mothers that seek to maintain breastfeeding, confirming previous findings where work policies and support influence mothers breastfeeding decisions (Abekah‐Nkrumah et al., 2020). In contrast, the participants who breastfed for longer did not regard breastfeeding in public or returning to work as an impediment. This could be due to the favorable breastfeeding environment in their workplace, albeit they mentioned that they do not have an adequate facility to express their milk. Also, these mothers see breastfeeding as natural/normal and reported to have understood this because of their participation in antenatal classes.

Family support, from grandparents, a partner, and extended family, is an essential component in a mother's decisions and practices. In this study, more senior family members (such as parents and mothers‐in‐law) were perceived to offer less support. Mothers were more likely to regard such advice as negative, hurtful, and confusing. Although mothers in this study did not stop breastfeeding due to unsupportive senior relatives, some introduced formula or water earlier than planned because of the perceived pressure. This advice affected breastfeeding mothers' experiences because it undermined their decisions (rather than supporting them). Several mothers described feeling that they could not trust their parents and expressed discomfort for having to defend their breastfeeding decisions. This could be due to a lack of knowledge, cultural beliefs, and past experiences of infant feeding. This confirms previous findings where the grandmother's opinion can outweigh the mother's opinion and even that of the pediatricians (Andrew & Harvey, 2011).

Breastfeeding promotion campaigns have a role in improving education surrounding breastfeeding practices in Panama. In contrast, participants reported that their partner often provided practical and emotional support. Their support for breastfeeding was perceived to be based on the health benefits for the baby and the financial cost of infant formula. Even though mothers reported feeling supported by their partners, some mentioned that their partners did not like when they had to breastfeed in public and asked them to cover up. The same mothers were the ones who introduced formula before 6 months. Consistent with previous research, partners' opinion about breastfeeding in public represent an obstacle for breastfeeding (Bueno‐Gutierrez & Chantry, 2015). Conversely, mothers who were able to maintain EBF mentioned that the most significant support came from their husbands, and when problems related to breastfeeding arose, the husbands themselves encouraged the continuation of breastfeeding. This behavior was correlated to those partners who had a more positive attitude toward breastfeeding in public.

While support from healthcare workers, such as pediatricians, gynecologists, and nurses, positively impacts breastfeeding establishment and duration (Khasawneh, 2016), in this study, such interventions did not influence decisions and practices regarding infant feeding in the antenatal period. Mothers in this study expressed their dissatisfaction regarding healthcare practitioners at the prenatal and postnatal period. They struggled with breastfeeding in the first weeks and felt that health professionals had not equipped them with information and support to solve these problems, leaving them feeling stressed about being unable to breastfeed. In addition, mothers were not content with the type of support they received in the hospital after the delivery by the nurses. These findings appear to support those of Theodorah and Mc'deline (2021). A study conducted in Panama by Rodríguez Diaz et al. (2014) found that the knowledge of healthcare professionals about the existence of policies or written regulations on the protection, promotion, encouragement, and support of breastfeeding was limited. Less than 40% of the healthcare works were aware of the WHO/UNICEF Baby‐Friendly Hospital Initiative; Law 50 of 1995 of Panama, which protects and promotes breastfeeding, or about the main points of the International Code of Marketing of Breast milk Substitutes. In a case study of Leon, Nicaragua, Safon et al. (2017) emphasized the need for integrated community support and the strengthening of regulation in respect to the marketing of infant formula and substitutes.

A distinctive finding of this study is that EBF mothers received help from a lactation consultant in the pre‐ and postnatal period. They reported that this help had been indispensable. It should be noted that in Panama there are no educational programs on breastfeeding before or after hospital discharge which contributes to the early abandonment of breastfeeding (Maguire et al., 2020). A lactation consultant must be obtained by the mother's own means and are not included in the public system. One of the mothers who could not maintain breastfeeding mentioned that such support was one thing that she lacked to overcome the barriers that arose. These views and experiences are consistent with the findings of Khasawneh (2016), in that support from healthcare workers, such as lactation consultants, in the first weeks after delivery has shown a positive impact on the establishment and duration of breastfeeding. A study conducted by Rollins et al. (2016) suggested that even with the nurses' guidance at the hospital, mothers were still presented with barriers that hindered breastfeeding, and that in most cases these can be resolved through individual counseling that occurs face to face and lasts more than 2 days after postpartum. This finding becomes important since having a lactation consultant is not the norm and this service is not always readily available and accessible either for economic reasons or due to a lack of awareness. These results fall within the second level of the socioecological model (social level), highlighting the role and importance of family, friends, healthcare providers, and other social support systems on mothers' behavior.

The main limitations of this study were the relatively small number of interviewees (*n* = 7) and single recruitment site. Greater participant diversity and a comparative element (with recruitment from multiple urban sites or from urban and rural sites) would have expanded the breadth of analysis. However, given the resource and time constraints for the project, an exploratory study based on a single site was most appropriate.

## 5 CONCLUSION

Although most of the mothers in this study acknowledge the benefits of breastfeeding for the infant's health, this does not necessarily guarantee the continuation of the practice. Mothers' desires represent only a small part of the decision‐making process. Family, workplace, and healthcare support were the main factors that influenced infant feeding decisions. Consistent with the socioecological model, the findings of this study suggest that individual, relationship, community, and societal aspects explain a mother's infant feeding decisions. For the mother to make an informed decision and feel confident, antenatal and postnatal education are needed. Moreover, it is a priority to provide educational interventions on the health benefits for the child and to expose the risks of artificial feeding and prepare her to solve the practical problems that arise with breastfeeding. It is important to include grandmothers and partners in such education efforts to help them provide adequate support and avoid harmful advice. Finally, more widely, adhering to progress toward increasing breastfeeding to promote the health of children in their first years of life requires increased awareness among the general population to phase out the stigma around breastfeeding.

## AUTHOR CONTRIBUTIONS

**Danae De La Cruz:** Conceptualization (equal); data curation (equal); formal analysis (equal); investigation (equal); methodology (equal); project administration (equal); writing – original draft (equal); writing – review and editing (equal). **Richard Lee:** Conceptualization (equal); data curation (equal); formal analysis (equal); methodology (equal); supervision (equal); writing – original draft (equal); writing – review and editing (equal). **Justine Gallagher:** Writing – original draft (supporting); writing – review and editing (supporting).

## CONFLICT OF INTEREST STATEMENT

The views expressed here are those of the authors and not necessarily those of the Ministry of Health, Republic of Panama.

## ETHICS STATEMENT

The authors declare that they have no conflicts of interest. Ethical approval for this study was granted by the Northumbria University Faculty of Health and Life Sciences Ethics Committee on February 17, 2021 (Submission Ref: 28851). Informed consent was obtained from all participants.

## Data Availability

The data are not publicly available due to privacy and ethical restrictions. Consent was not sought from participants for the sharing of whole interviews and the dataset to respect their anonymity, though sections of the interview transcripts are presented in the article.
